# The gendered effect of an overwork climate and high personal standards for work–home conflict during the pandemic

**DOI:** 10.1177/0143831X231167497

**Published:** 2023-04-25

**Authors:** Arūnas Žiedelis, Jurgita Lazauskaitė-Zabielskė, Ieva Urbanavičiūtė

**Affiliations:** Institute of Psychology, Organizational Psychology Research Center, Vilnius University, Lithuania

**Keywords:** Gender, high personal standards, overwork climate, work–home conflict

## Abstract

Although working from home and various other forms of flexible work are often presented as measures to strengthen work–life balance, research depicts a less optimistic picture. Previous research has shown that the impact of telework on work–home conflict is controversial, depending on various factors that are also frequently gender-specific. In this study, the authors evaluate and compare the effects of external expectations (i.e., an organizational overwork climate) and internal expectations (i.e., high personal standards) on changes in work–home conflict between working men and women during the COVID-19 pandemic in Lithuania. Both types of expectations were associated with difficulties reconciling work and private life. Due to their interactions with stereotypical gender roles, organizational expectations encouraging overtime work had a more pronounced effect on male employees. Results suggest that an overwork climate within organizations is a problem not only for employees’ well-being but also poses a risk to gender equality in work and private life.

## Introduction

The COVID-19 pandemic and the lockdown implemented to limit its spread have significantly changed the ways of living and working. In Europe, one out of two employees was required to start handling their work responsibilities while staying at home, and for many, this was their first attempt to work out of the office (Eurofound, 2020). Working from home is sometimes presented as a means of achieving a greater work–home balance (European Commission, 2010). However, existing research suggests that this effect is not unequivocal and depends on various organizational and individual factors (Eurofound and the International Labour Office, 2017). Moreover, transitioning from regular office work to working from home requires preparation, which was hardly possible due to the turbulent circumstances of the pandemic. For this reason, not all employees reported a decreased work–home conflict; for some, it remained the same or even increased (Schieman et al., 2021; Vaziri et al., 2020).

During the pandemic, many organizations could not adequately prepare for the transition to teleworking and thus take care of the well-being of workers and their families (Chen, 2021). Therefore, various social and psychological factors that shape perceptions of the role of work had an even more significant impact on how work is now performed and coordinated with other areas of life. For example, although employers have not openly expressed such a requirement, some employees report having started working longer hours during the pandemic, which creates additional challenges for balancing work and non-work needs (Deole et al., 2021). In addition to that, female employees reported more difficulties in combining work and private life and were more likely to reduce their work hours because of increased responsibilities at home than their male colleagues (Collins et al., 2021; Eurofound, 2020), which means that the context of a pandemic affects male and female employees differently. In this study, we delved into how high personal standards and organizational norms to work overtime (i.e., an overwork climate) predicted changes in work–home conflict and whether this effect was equal for men and women. As a result, we made the following contributions to the existing literature.

First, by employing a time-lagged analytical approach, we uncovered how external and internalized expectations of working hard (i.e., an overwork climate and high personal standards) predicted an increase in work–home conflict during the pandemic. The research on work–home interaction has been criticized for an overreliance on resource-based theories that place the greatest emphasis on stable situational characteristics such as job demands (for a recent review, see Allen and French, 2023). Instead, using the theoretical approach of role theory, we show that both external and internalized expectations that shape demands for the work role are equally important. Additionally, we demonstrated that high personal standards, which are sometimes considered a marker of adaptive perfectionism, predict higher work–home conflict. In this way, our results support the differential susceptibility hypothesis (Belsky and Pluess, 2009; Gaudreau et al., 2018), according to which the so-called adaptive perfectionism can become maladaptive for the pursuit of high standards in unfavorable circumstances.

Second, our study showed that work role expectations were not equally important in predicting work–home conflict among working men and women. Recently, Allen and French (2023) noted that work–home conflict research had been more focused on breadth (i.e., looking for a variety of predictors and relationships) than depth (i.e., trying to understand when and why the strength of certain relationships varies). In contrast to this trend, we not only analyze the predictive importance of work role expectations but also explore how the salience of expectations differs between genders. In addition, when examining differences in a work–home conflict between men and women, Shockley et al. (2017) point to the potential importance of cultural norms, yet suggest focusing on smaller-scale factors such as organizational culture. Our study responds to this call by showing that organization-based overtime expectations (i.e., an overwork climate) were more relevant for men and almost irrelevant for women’s work–home conflict. This allows us to identify men as a group of employees for whom – most likely due to the still prevalent male breadwinner stereotype (Eagly and Wood, 2012) – the normative climate in the organization is particularly significant when balancing work and personal life. In such a way, the overwork climate promoted in organizations poses problems not only for the well-being of employees but also for efforts to ensure gender equality in the workplace and outside of work.

## Work–home conflict during the pandemic

When it comes to balancing work and home commitments, one of the key contextual factors in recent years has been the COVID-19 pandemic, followed by the lockdown and subsequent necessity to work from home, which had an equivocal effect on work–home conflict. For some employees, it was an opportunity to balance different role expectations more efficiently. As a result, they were relieved from time-consuming activities such as commuting to work and enjoyed more flexible boundaries between work and non-work domains (Thompson et al., 2022; Vaziri et al., 2020). However, for others, challenges related to the work–home interface have only increased (Allen et al., 2021; Kerman et al., 2022; Vaziri et al., 2020). These results contrast with the pre-pandemic scholarship in remote working, which is sometimes presented as a promising solution aimed at helping employees balance their work and non-work responsibilities (European Commission, 2010). Claims about the universal benefits of remote working are not well supported by research data, which show that the cost and benefits of flexible work might depend on individual and contextual factors (Eurofound and the ILO, 2017; Gardner et al., 2021). Therefore, it is difficult or even impossible to answer the question about the impact of working from home on work–home conflict without considering other factors that shape the interaction of different life domains (such as gender-related role expectations).

Work–home conflict describes an inter-role conflict, which occurs when what is expected of employees by colleagues, managers, the organization, society, or the employees themselves becomes incompatible with role expectations in the non-work domain (Greenhaus and Beutell, 1985). According to role theory (Biddle, 1979; Eagly and Wood, 2012), individuals taking a particular position (e.g., employee, family member) encounter certain expectations (i.e., role pressures) that guide their behavior and form specific behavior patterns called roles. For example, at work, the person fulfills the role of an employee in line with the corresponding behavioral expectations attached to this role. In contrast, the same person must attend to a different set of expectations related to their non-work roles at home. Although most people have multiple roles in multiple contexts, for many, the central roles of their lives are enacted within the work and home domains (Greenhaus and Powell, 2012). Both work and home roles compete for limited resources such as time, attention, and effort and sometimes require different patterns of behavior.

## Overwork climate as an external work role expectation

The major difference between roles on stage and in society is that the latter are shaped not by defined scenarios but by variously articulated expectations. Basically, a role is a set of behavioral expectations assigned to a certain group or category of people that specify the boundaries of desirable and acceptable behavior (Anglin et al., 2022; Biddle, 1979). In other words, when assessing the extent to which the observed behaviors are acceptable and welcome, the decision is made taking into account the expectations of the role being performed at the time. In this way, social roles affect cognitions, affects, and behaviors, motivating certain forms of behavior and shaping evaluations of one’s own behavior and that of others (Sluss et al., 2011).

Behavioral expectations that describe role characteristics and serve as standards to evaluate the appropriateness of behavior have both social (external) and psychological (internalized) aspects. External role expectations reflect a socially shared consensus on acceptable behaviors in a particular group or society (Anglin et al., 2022). For instance, organizations may have expectations that their employees will be ready to work on weekends, society may associate the role of the mother with an exclusive priority for the needs of the family, etc. Such external pressures shape behavior because failure to meet them can lead to negative consequences such as rejection or condemnation.

It has recently been proposed that organizations differ in how much they expect their employees to perform overwork (Mazzetti et al., 2014, 2016). Such propositions bring to mind the sociological concept of greedy organizations as social institutions that demand total commitment from their members, requiring them to prioritize the organization’s interests, even despite the responsibilities arising from other social roles (Coser, 1967). Although the time and effort an employer may demand from employees are legally regulated and set out in employment contracts, various normative expectations influence the commitment to the organization that is encouraged and considered normal and the extent to which personal life should be sacrificed for work. For example, in organizations with a high overwork climate, employees perceive that they must work more than is legally required to be promoted and valued by their supervisors. Moreover, high work investment norms are embedded in specific work procedures and practices (for example, arranging meetings late in the afternoon or contacting employees after regular working hours) and modeled by other organization members (Mazzetti et al., 2016). Most importantly, an overwork climate regulates employees’ behavior both during and after working hours. Mazzetti et al. (2014) have found that an overwork climate encourages heavy work investment, especially in combination with such individual factors as achievement motivation and perfectionism.

The pandemic context should also be considered in this regard, as contextual factors such as the unemployment rate and increased connectivity through telecommunication technologies encourage organizational greediness (Sullivan, 2014). During the pandemic, the number of employees working from home via communication technologies increased dramatically (Eurofound, 2020). Such changes are known to blur or even erase the boundaries between work and non-work, allowing organizations to reach their employees more easily after official working hours (Otonkorpi-Lehtoranta et al., 2022). Moreover, as unemployment skyrocketed because of the lockdown and the reduced demand for the workforce, it became easier for organizations to impose even unpopular norms both due to the increased pool of jobseekers (and subsequently increased job insecurity) and the opportunity to justify overwork demands by unprecedented economic circumstances (EU-OSHA, 2021). Thus, we propose our first hypothesis:

*H1*: Overwork climate will predict an increase in work–home conflict over time during the pandemic.

## High personal standards as internalized expectations

Although external expectations significantly influence the content of the work role, individual factors may be no less critical, as they determine how the role will be perceived and how important it is for the individual to meet the expectations of the role. The more recent symbolic interactionist role theory perspective emphasizes the importance of role identity as the level at which role expectations are interpreted, internalized, and followed (Sluss et al., 2011; Stryker & Serpe, 1994). This perspective emphasizes that although structurally individuals may face analogous external expectations, the perception and importance of those expectations are not necessarily the same for everyone. Individuals may differ in their internalization of role expectations, which affects their behavior even after external pressures are removed (Stryker, 2001).

One known factor that encourages working beyond standard requirements, even in the absence of external pressure and thus increasing work–home conflict, is perfectionism. Navigating between the conflicting expectations of work and home domains requires the ability to negotiate and make compromises since being a perfect employee is hardly, if ever, compatible with being a perfect spouse, parent, or friend (Peters and Blomme, 2019). Such ability might be affected by perfectionistic dispositional factors, encouraging the maintenance of high personal standards (Mitchelson, 2009). Employees prone to perfectionism are characterized by a tendency to set high internal standards: that is, to strive to perform tasks remarkably well. They may also tend to (though not necessarily) experience a mismatch between high internal standards and their actual accomplishments (Stoeber et al., 2018).

Adaptive and maladaptive types of perfectionism are often distinguished. High personal standards characterize both types, but in the case of maladaptive perfectionism, self-criticism due to the inability to achieve set goals is also observed (Deuling and Burns, 2017). However, recent studies examining the differential susceptibility hypothesis point out that the adaptability of high personal standards depends significantly on whether the external environment is favorable for achieving goals (Belsky and Pluess, 2009; Gaudreau et al., 2018). In other words, the so-called ‘adaptive perfectionists’ function better than individuals without such dispositions when the environment is supportive. But, on the other hand, they also react more sensitively (negatively) to circumstances unfavorable to high aspirations. As a result, the distinction between adaptive and maladaptive perfectionism may have little relevance in less-than-optimal circumstances (Gaudreau et al., 2018).

In this study, we state that the high personal standards dimension of perfectionism is important for changes in work–home conflict during the pandemic. We base our reasoning on the following rationale. First, people who demonstrate this trait are more susceptible to the influences of the environment for better or for worse (Gaudreau et al., 2018). For example, previous research has shown that so-called adaptive perfectionism, characterized by high personal standards, is related to more intense experiences of pride when reacting to achievements, and shame, when confronted with failures (Stoeber and Yang, 2010; Stoeber et al., 2008). Thus, the same high personal standards might be adaptive and maladaptive, depending on the external context. Given that the lockdown has not been favorable to many workers in achieving their goals, high personal standards may likely function as a risk factor in this context.

Second, perfectionism may hinder task performance, as it encourages the prioritization of high performance standards, even though it costs a disproportionate amount of time and effort, thus reducing the ratio between input and output (Gaudreau, 2019). Optimizing task performance standards can be especially important during the pandemic when conditions to meet exceeding standards are not necessarily ideal. For example, achieving the same results in the face of communication technology problems may require additional resources (such as time and effort) that are not proportionate to the outcome.

Third, returning to role theory, high personal standards in the work environment function as internalized expectations for the work role (Stryker, 2001). Although the expectation to perform work tasks to the best of one’s ability may be externally supported, employees who set high personal standards for themselves will, by definition, tend to pursue higher goals and devote more time and effort to them even in the absence of external pressures (Gaudreau et al., 2018). Having in mind that high internal standards can lead to higher internalized expectations for a work role, greater sensitivity to external pressures to perform, and difficulties in adjusting one’s own goals according to the circumstances, we speculate that they should be related to an increase in work–home conflict during the pandemic and present the following hypothesis:

*H2*: High personal standards will predict an increase in work–home conflict over time during the pandemic.

## The moderating effect of gender

Although, culturally, work–home conflict is often considered a more pressing problem for women, some recent studies show that employees of both genders face difficulties in combining work and personal life (Shockley et al., 2017). Similarly, internal and external pressures to work at the expense of one’s family’s well-being can be experienced by both men and women. However, as the impact of these pressures is based on normative influence, it may be enhanced or weakened by society’s gender-specific normative expectations. Conventional gender stereotypes attribute the role of the family’s financial provider (i.e., the ‘breadwinner’) to men and the role of caretaker of various emotional and household needs (i.e., the ‘caregiver’) to women (Eagly and Wood, 2012). These differences in role expectations stem from the centuries-old sex-based division of labor (Wood and Eagly, 2012). In most pre-industrial societies, due to being the primary caregiver for infants, women, in general, were excluded from tasks that required uninterrupted periods of activity away from home and instead performed activities that were more compatible with reproduction and childcare. Such division influences power relationships and is responsible for differences in gender role expectations by forming the male ‘breadwinner’ and female ‘caregiver’ cultural norms (Wood and Eagly, 2012). Although socioeconomic conditions changed dramatically when women entered the workforce, and the division of labor became far less extreme, the traditional gender expectations retain a strong behavioral influence on employees and their employers (Bear and Glick, 2017). Even though most employees come from dual-earning families, providing for the family is still a component of normative masculinity (Kanji and Samuel, 2017). For similar reasons, women spend more time on household chores than men (Cerrato and Cifre, 2018).

Working from home is often presented as a means of reconciling work and private life, thus equalizing opportunities for men and women (European Commission, 2010). However, previous studies warn that this might not address gender issues but instead allow the exploitation of women in both work and domestic areas (Chung et al., 2021; Sullivan and Lewis, 2001). In particular, men and women take advantage of the opportunities offered by working from home in different ways, influenced by gender stereotypes. Women spend more time on household chores while working remotely. Meanwhile, men working remotely are likelier to work more hours (Chung and van der Horst, 2020). In this way, teleworking opportunities do not reduce gender gaps but exacerbate them.

In addition, differences between male and female gender stereotypes become important when employees face internal and external pressures to work harder. In the case of women, the ‘caregiver’ cultural norms encourage them to have more responsibilities at home (Cerrato and Cifre, 2018; Chung and van der Horst, 2020; Eurofound, 2020), thus high internal standards may create greater work–home conflict when work tasks have to be reconciled with high family demands. On the other hand, the stereotypical role of the ‘caregiver’ encourages women to prioritize family needs over work (Eagly and Wood, 2012) and, paradoxically, can mitigate the effects of overwork norms. Indeed, a study by Cha (2013) found that in organizations that promote overwork and overtime performance by rewarding it with promotion, women are less likely to respond to such norms and, consequently, have fewer career advancement opportunities than men. Moreover, during the COVID-19 pandemic, women were more likely than men to reduce their workload as it became more difficult to reconcile it with family needs (Collins et al., 2021).

In the case of men, the situation is reversed. The male ‘breadwinner’ stereotype encourages the prioritization of work, career, and financial support for the family (Eagly and Wood, 2012). Thus, it resonates more strongly with and reinforces the organization’s overwork expectations (Kanji and Samuel, 2017). As a result, male employees are more likely to sacrifice family for the sake of work, report working more than they would like, and more likely to be stigmatized if they have to use work–home management policies, such as parental leave (Cha, 2013; Kanji and Samuel, 2017; Vandello et al., 2013). On the other hand, the same stereotype means that men have fewer responsibilities at home (Cerrato and Cifre, 2018), leading to better opportunities to achieve high internal standards without sacrificing duties at home. It should also be noted that men experienced less work–home conflict during the pandemic, even though the gender gap in work hours has significantly increased (Collins et al., 2021; Eurofound, 2020).

Thus, we propose our third hypothesis:

*H3*: Employee’s gender will moderate the effect of high personal standards and overwork climate on the change in work–home conflict:
*H3a*: The effect of high personal standards for the increase in work–home conflict over time during the pandemic will be more salient among female employees.
*H3b*: The effect of overwork climate for the increase in work–home conflict over time during the pandemic will be more salient among male employees.

## Methods

### Sample and procedure

To measure the change in work–home conflict, we collected two waves of data in November 2020 and March 2021. Both study waves were carried out within the period of lockdown to limit the spread of coronavirus, when most employees were forced to work from home. The first lockdown was introduced in Lithuania in March 2020, and there was a brief return to a more or less normal lifestyle before another lockdown was reintroduced in November 2020, which lasted until June 2021. Therefore, the first study wave was conducted at the very beginning of the second lockdown, and the second wave took place four months later.

The participants were recruited through network sampling with the help of student research assistants and received no compensation for participation in this study. A heterogeneous sample of 883 Lithuanian employees completed the questionnaire of the first study wave and were invited to participate in the second wave. The respondents were asked to express their consent to be contacted again by providing their email addresses. In such a way, we received 375 emails and 235 respondents participating in both study waves (response rate: 62.7%). We compared those who participated in both study waves with those who dropped out regarding sociodemographic and psychological factors. This analysis revealed that participants who dropped out were somewhat younger (31.8 vs. 35.9 years; *t* = −3.339, df = 339, *p* = .001) and less likely to work in the public sector (32.1% vs. 42.6%; χ^2^ = 4.009, df = 1, *p* = .045), but in all other cases, the differences between the two groups were non-significant. For this reason, we used a total sample of 375 respondents for further analysis and used the full information maximum likelihood (FIML) estimator to account for missing cases.

The sample consisted of 89 males and 286 females aged 18 to 65 (*M* = 34.4, *SD* = 12.4). Of these, 230 participants (61.3%) worked in the public sector, and 145 (38.7%) worked in the private sector. Most participants had a full-time job (81.3%) and worked from home at least part of the time (92.5%). On average, participants were working remotely 4.3 days a week.

### Measures

Respondents were asked to provide demographic data (gender, age, having minor children, public versus private sector, number of days working from home) and fill out a questionnaire including items to assess work–home conflict, high personal standards, and overwork climate. All measures were translated from English to Lithuanian by applying a back-translation procedure to ensure that all items were consistent with their original meaning (Brislin, 1980).

*Work–home conflict* was assessed during both study waves by the negative work–home interaction subscale obtained from the SWING questionnaire (Geurts et al., 2005). The subscale consists of four items, rated on a five-point Likert-type scale, ranging from 1 – never to 5 – always/almost always. A sample item is: ‘How often does it happen that your work schedule makes it difficult for you to fulfill your domestic obligations?’

*High personal standards* were measured during the first study wave by four items obtained from the Revised Almost Perfect Scale (Slaney et al., 2001). These items were rated on a seven-point Likert-type scale, ranging from 1 – totally disagree to 7 – totally agree. A sample item is: ‘I set very high standards for myself’.

We measured employee’s perception of the *overwork climate* during the first study wave with the eight-item Overwork Climate Scale (Mazzetti et al., 2014). All items were rated on a five-point Likert-type scale, ranging from 1 – totally disagree to 5 – totally agree. A sample item is: ‘Almost everybody expects employees to perform unpaid overtime work’.

### Data analysis

We modeled latent change score (LCS) analysis with IMB AMOS 20.0 to test our study hypotheses. LCS analysis is a flexible modeling technique based on a structural equation modeling (SEM) framework and designed specifically to address questions related to the determinants of unequal intra-individual changes across measurement occasions (Klopack and Wickrama, 2020). As the name implies, in LCS models, change is represented by a latent variable instead of a simple comparison of observed scores (i.e., X_t_ – X_t-1_). The latent variable is then used as a dependent variable to estimate the effect of independent variables on longitudinal change (Matusik et al., 2021). The main advantage of LCS modeling (compared to simple subtraction of observed scores) is that it accounts for the measurement error; thus, measurement reliability is increased.

All measures were inspected for multigroup (male and female groups) measurement invariance before testing the hypotheses. Moreover, as work–home conflict was measured twice, we tested both the multigroup and longitudinal invariance of this measure. We tested for configural and metric invariance (Putnick and Bornstein, 2016) to establish the equivalence of model form and factor loadings at both time points and for both genders. Metric invariance was observed if the Comparative Fit Index (CFI) decreased by less than .01 and the Root Mean Square Error of Approximation (RMSEA) increased by less than .015 after imposing constraints on factor loadings (Chen, 2007). For all three study measures, the models with fixed equal factor loadings among groups fit data equally well as the configural models (see Table A1 in the Appendix for more details). Thus, metric invariance was observed and further structural models were built, constraining factor loadings to be the same for both genders.

**Figure 1.**
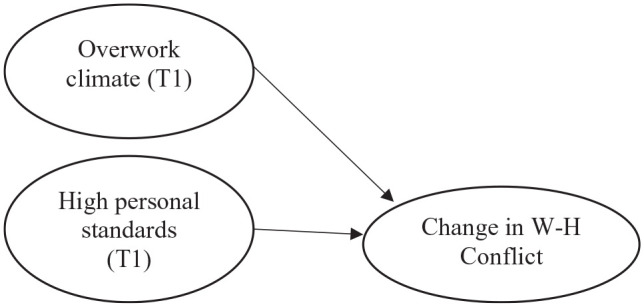
Baseline model. *Note.* For the sake of parsimony, observed variables, error variances and correlations between exogenous variables are not presented.

We tested our baseline model (see Figure 1) on a full sample to estimate the effects of personal standards and overwork climate on the change in work–home conflict four months later. Hypotheses regarding these effects (H1 and H2) were supported if model fit was acceptable and the regression weights were positive and significant. Moreover, we used a multigroup modeling approach to test the moderating effect of employee’s gender. More specifically, alongside the baseline multigroup model, we separately constrained each regression path from overwork climate and high personal standards to be equal for both genders and compared these models with the unconstrained model. Hypothesis H3a would be supported if the regression weight of the path from high personal standards to change in work–home conflict was stronger among females, and the multigroup model with constrained equal paths fit data worse than the baseline model. Similarly, H3b would be supported if the regression weight of the path from overwork climate to change in work–home conflict was higher among males, and the multigroup model with constrained equal paths fit data worse than the baseline model.

## Results

Associations between the main variables are presented in Table 1. Employee’s age, minor children at home, sector, and the number of days working from home had only trivial correlations with the main study variables (*r* < .2), therefore they were not included in further analysis. As expected, work–home conflict had high autocorrelation across the two measurement points and was positively associated with high personal standards and overwork climate. Moreover, while on average the levels of work–home conflict remained quite similar between the two study waves (*t* = −1.589, df = 235, *p* = .113), the change score had a significant variance within the sample (*σ2* = 0.591, *SE* = 0.080, *p* < .001). This means that intra-individual changes were somewhat balanced within the sample: that is, work–home conflict may have increased for some employees but decreased for others. It might also be relevant to note that high personal standards and overwork climate showed only weak and non-significant correlation, which supports the assumption about the different nature of external and internalized role pressures.

**Table 1.** Descriptive statistics and correlations between the study variables.

	*M*	*SD*	1	2	3	4	5	6	7	8
Age	34.4	12.4								
Children	1.8	0.4	−.17**							
Sector	1.4	0.5	.11*	.04						
NDWH	4.3	1.4	−.03	.05	.02					
HPS	5.5	1.3	−.17**	.04	−.05	.02	(.94)			
OW	2.6	0.8	.05	.03	.08	.03	.08	(.85)		
WHC^1^	2.6	1.0	.04	−.04	.09	.01	.15**	.46***	(.86)	
WHC^2^	2.7	1.0	.13*	.03	.05	.01	.26***	.45***	.64***	(.87)
Gender	1.8	0.4	.09	.08	.03	.00	−.01	.10	.09	.07

*Notes.* Gender (1 = male, 2 = female), Children (1 = has underage children, 2 = does not have underage children), sector (1 = public, 2 = private), NDWH – number of days working from home (per week), HPS – high personal standards, OW – overwork climate, WHC – work–home conflict (index number indicates the study wave). Cronbach’s alpha coefficients are presented on the diagonal. *p < .05; **p < .01; ***p < .001.

To test H1 and H2, we estimated the fit indices and inspected the regression paths of our baseline model (see Figure 1), with male and female employees included as a single group. Fit indices showed that the baseline model had an acceptable fit to the data (χ^2^ = 555.514, df = 163, *p* < .001; RMSEA = .080, CFI = .904). Furthermore, both high personal standards (B = 0.111, *SE* = 0.041, *p* = .007) and overwork climate (B = 0.233, *SE* = 0.096, *p* = .016) had a significant effect on the latent change score in work–home conflict. Thus, our first two hypotheses, regarding the effect of high personal standards and overwork climate on the change in work–home conflict, were supported.

To test H3 (the moderating effect of gender), we modeled a multigroup baseline model for males and females separately and included additional constraints. More specifically, we constrained the paths from high personal standards and overwork climate to the change in work–home conflict factor to be equal between genders. The regression weights and fit indices of these models (including changes in chi-squared statistics compared to the baseline model) are presented in Table 2.

**Table 2.** Regression weights and fit indices of compared multigroup models.

	Models		
	Baseline multigroup model	Equal HPS effect between genders	Equal OW effect between genders
Regression weights for women			
HPS → ΔWHC	.09(.04) / .17*	.11(.04) / .21**	.09(.04) / .16*
OW → ΔWHC	.12(.10) / .13	.12(.10) / .12	.19(.10) / .19*
Regression weights for men			
HPS → ΔWHC	.24(.11) / .29*	.11(.04) / .14**	.16(.11) / .20
OW → ΔWHC	.74(.26) / .44**	.63 (.25) / .38*	.19(.10) / .12*
Model fit measures			
χ^2^	773.47***	774.99***	777.51***
df	339	340	340
Δχ^2^(Δdf)		1.52(1)	4.04(1)*
RMSEA	.06	.06	.06
CFI	.90	.90	.89

*Notes.* HPS – high personal standards, OW – overwork climate, ΔWHC – change in work–home conflict. When presenting regression weights, unstandardized coefficients are provided before the backslash, standardized coefficients are provided after the backslash, and standard errors are presented in parentheses. *p < .05; **p < .01; ***p < .001.

Contrary to our expectations, after constraining the path from high personal standards to change in work–home conflict to be equal for men and women, the chi-squared difference was non-significant (Δχ^2^ = 1.52, Δdf = 1, *p* = .218), which means that the constrained model did not differ from the unconstrained model. Moreover, the results of the baseline model revealed that the relationship between high personal standards and changes in work–home conflict was somewhat higher (although non-significantly) among males than females. Thus, our H3a hypothesis regarding the moderating role of gender in the relationship between high personal standards and change in work–home conflict was not supported.

Finally, after constraining the path from overwork climate to change in work–home conflict to be equal for both genders, the model fit decreased significantly compared to the baseline model (Δχ^2^ = 4.04, Δdf = 1, *p* = .044). Moreover, the regression path from overwork climate to the change in work–home conflict in the baseline model was significant only among males but not females. Thus, H3b regarding the moderating effect of gender on the effect of overwork climate on the change in work–home conflict was fully supported.

## Discussion

Before the pandemic, work and non-work roles were usually performed in different locations. Thus, the need to accommodate these roles while staying in the same limited space during the lockdown was a unique challenge. The experiences of many employees confirm that working from home provides both opportunities and challenges for balancing expectations from different roles (Allen et al., 2021; Kerman et al., 2022; Vaziri et al., 2020). Due to various internal and external influences, not everyone was equally successful in dealing with work–home conflict while working outside the office. Our research aimed to evaluate the effect of an overwork climate and high personal standards on the changes in work–home conflict during the second lockdown (i.e., four months) among male and female employees.

The two-wave research strategy provided an opportunity to assess not only differences between employees but also temporal changes within the same individuals. Our study sheds light on the somewhat equivocal earlier findings linking working from home to work–home conflict. Although, on average, the levels of work–home balance seemed relatively stable during both study waves, there was a considerable variance in individual change trends. In other words, while some employees could better balance expectations from different domains, this inter-role conflict had only increased for others. These results are consistent with data from previous studies showing that working from home can either help or hinder balancing work and home responsibilities (Allen et al., 2021; Vaziri et al., 2020). Moreover, they underline the importance of understanding what individual and contextual factors explain unequal changes in work–home conflict when employees are forced to work from home for a prolonged period.

In this study, we identified factors related to work role expectations (external and internalized) predicting increased work–home conflict during the pandemic. More specifically, our findings revealed that one of the hallmarks of perfectionism – high personal standards – was associated with increased work–home conflict. While high personal standards do not necessarily mean that an employee is characterized by non-adaptive perfectionism, our data are consistent with the differential susceptibility hypothesis stating that even adaptive perfectionists face more negative consequences if the environment is not conducive to goal achievement (Gaudreau et al., 2018). Our results also complement the studies that have established a distinction between different forms of perfectionism (Gaudreau, 2019; Stoeber et al., 2018). The fact that even adaptive perfectionists, due to their sensitivity to adverse contextual factors, may experience greater work–home conflict means that the distinction between adaptive and non-adaptive perfectionists might only make sense in favorable circumstances. Although future research should test this assumption with other work-related consequences, it might be that in the context of the pandemic, when sudden changes in working conditions make it difficult to achieve goals in a usual way, any form of perfectionism may hinder work–home balance.

Our results also revealed that increased work–home conflict during the pandemic might be predicted not only by internalized pressures to perform better than usual (i.e., high personal standards) but also by the corresponding normative expectations within the organization (i.e., an overwork climate). Previous research has shown that an overwork climate encourages heavy work investment by promoting norms to put extra time and effort into work through various policies, practices, and procedures (Mazzetti et al., 2014; Schaufeli, 2016). Our results revealed that external expectations for excessive work remained relevant even after the transition to remote work. It is also important to note that an overwork climate was moderately correlated with the initial work–home conflict. Thus, in organizations that encourage overwork, employees not only had higher initial levels of work–home conflict, but it was also more likely to increase during the pandemic.

Finally, the most intriguing finding of this study concerns the interaction between work role expectations and gender. We hypothesized that the importance of work role expectations would not be the same among women and men. In other words, we speculated that external expectations of working harder during the pandemic would be more salient among males since they correspond to the cultural norm of the male ‘breadwinner’. On the other hand, internal expectations to achieve high standards were thought to increase the risk of experiencing work–home conflict for women due to their greater responsibilities at home. Our results only partially confirmed these considerations. More specifically, although the effect of high personal standards on work–home conflict was similar for both genders, male employees were more affected by an overwork climate. It is important to note that the mean levels of the main constructs of the study did not differ between genders (see Table 1), suggesting that unequal starting levels of the outcome variables among men and women cannot explain these results.

These results also contrast with the ideas expressed by Shockley et al. (2017) that stereotypical gender roles make it easier for men to balance work and home commitments. According to these authors, men are traditionally assigned the family breadwinner role, making it easier for them to simultaneously fulfill the demands of work and home by working hard. Our results signal the opposite trend: having a harder time resisting expectations to work more than they should, men face greater work–home conflict in organizations that support such expectations. Moreover, our results are consistent with a study by Cha (2013) that revealed that an overwork climate affects male and female employees differently. Despite all the progress made to ensure equal opportunities for men and women, masculinity is often associated with the financial maintenance of the family (Kanji and Samuel, 2017). In addition, such cultural attitudes give organizations additional leverage to project overtime expectations onto their male employees, thus increasing their work–home conflict while simultaneously limiting career opportunities for women (Cha, 2013).

Contrary to what is sometimes believed in popular culture, balancing work and home responsibilities is equally challenging for both genders. However, the reasons men and women experience conflict are not precisely the same (Shockley et al., 2017). And although women’s specific difficulties in balancing work and personal life receive more attention (e.g., Anglin et al., 2022), our study revealed at least one case where men are disadvantaged. Our data reaffirm, again, that organizations should avoid an overwork climate and should care about a work–life balance culture. It is worth noting that often initiatives aimed at helping to combine work and home obligations are focused on women raising children. Our study revealed that such initiatives should not bypass men, who, for cultural reasons, may face more significant difficulties in resisting the norms promoting work at the expense of the family.

### Limitations and future directions

Several study limitations should be acknowledged. First, the study conclusions should be generalized with caution due to the convenience sample. Second, in this study, we measured only the fact of having children, which may not have revealed the specific characteristics of parents raising children of different ages. For example, a recent Eurofound (2020) report found that work–home conflict has increased most among mothers raising children under 12 years of age. Thus, although our study has shown that having children was unrelated to the main phenomena we studied, future studies should consider the children’s age. Third, the confirmatory factor analysis of the overwork climate measure showed a poor fit of a single-factor structure. Fourth, our sample size was somewhat limited, and gender distribution was uneven, potentially resulting in a lack of statistical power to identify weaker relationships. More specifically, while we need to recognize that gender differences are more pronounced regarding employees’ experienced overwork climate, it is possible that high personal standards also affect men and women differently. Thus, future studies should aim to collect more extensive and balanced samples. Fifth, it is worth paying attention to the broader cultural context of the country where the study was conducted. According to the latest Global Gender Gap Index (World Economic Forum, 2022), Lithuania ranks 11th globally and is among the leading countries in ensuring gender equality. Although previous research findings do not support the idea that cultural egalitarianism influences differences in the work–home conflict between men and women (Shockley et al., 2017), the general lack of cross-cultural research calls for caution regarding the generalizability of the findings before testing them in diverse cultural contexts. Finally, our study revealed that high personal standards and an overwork climate were associated with a higher likelihood of increased work–home conflict. Still, the exact mechanisms for this effect are not clear. Drawing from role theories, we speculate that both personal standards and organizational norms function as expectations that shape the various work roles, thus encouraging employees to invest more time and effort into work, leaving fewer resources for the home sector. However, these assumptions need to be clarified in future studies.

## Conclusion

The pandemic and the lockdown have allowed many employees to test in practice the validity of considerations about the relationship between telework and work–home balance. As a result, it has become clear that the impact of working from home on reconciling different areas of life is not straightforward and depends on a variety of individual and organizational factors. Therefore, there is a long way to go to ensure that teleworking is beneficial for the well-being of employees and gender equality. At the very least, our research has shown that meeting high internal or organizational expectations in the face of unfavorable pandemic circumstances often means that family life will suffer. And this point is especially relevant for men who find themselves vulnerable due to outdated yet widespread cultural stereotypes.

## Appendix

**Table 1A.** Fit indices of measurement invariance models for main study measures.

Models	Fit indices		
	χ^2^ (df)	RMSEA	CFI
*Work–home conflict*			
Multigroup longitudinal configural invariance	96.529 (30)***	.077	.952
Multigroup longitudinal metric invariance	108.876 (39)***	.069	.949
*High personal standards*			
Multigroup configural invariance	87.761 (4)***	.237	.942
Multigroup metric invariance	88.038 (7)***	.176	.944
*Overwork climate*			
Multigroup configural invariance	246.250 (40)***	.118	.820
Multigroup metric invariance	253.635 (47)***	.109	.820

* *p* < .05; ***p* < .01; ****p* < .001.
